# An Extensive Case of Aplasia Cutis Congenita

**DOI:** 10.7759/cureus.63215

**Published:** 2024-06-26

**Authors:** Matthew D Rose

**Affiliations:** 1 Paediatric Intensive Care, Leeds General Infirmary, Leeds, GBR

**Keywords:** neurosurgery, plastic and reconstructive surgery, connective tissue disorder, scalp lesions, term neonate

## Abstract

Aplasia cutis congenita (ACC) is a rare connective tissue disorder that affects the epidermis, dermis, and subcutaneous tissue. Lesions can be small and benign or, in some cases, large and extensive. The lesions can be covered by a thin membrane, and depending on their location, they can make the underlying tissue vulnerable to damage or infection. This case report focuses on a term male infant born with extensive scalp defects and later diagnosed with ACC. The study will discuss the pathophysiology and classification of this disorder. This will provide clinicians with a recommended approach for the initial management of infants diagnosed with ACC in their practice.

## Introduction

While antenatal screening and imaging are improving year by year, infants are still born with unexpected congenital defects. Recognition of these conditions can help identify if they are benign or life-threatening and guide clinicians to further investigations in cases where the anomalies may be linked with genetic defects or syndromes. This study covers a term infant born unexpectedly with large defects to his head and diagnosed with aplasia cutis congenita (ACC). This is a rare condition causing congenital absence of the skin, affecting 1 in 10,000 births, and most commonly causing defects to a patient's scalp [1,2]. It can occur spontaneously or be associated with other congenital abnormalities or syndromes. It can also be associated with antenatal infections, such as varicella. There is no clear consensus on how best to manage the condition. Small defects heal naturally over time and form hairless scars, whereas larger, deeper lesions may require skin or bone grafting to repair. Due to the rarity of the disease and its wide range of presentations, input from multiple teams is often necessary to decide on treatment plans and ensure the best outcomes for a patient.

## Case presentation

**Figure 1:**
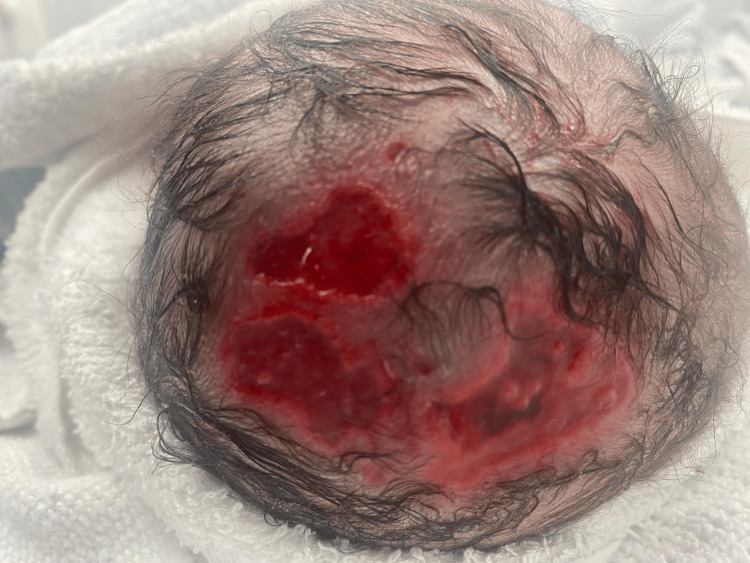
View of the lesions on the top of the affected infant's scalp

**Figure 2:**
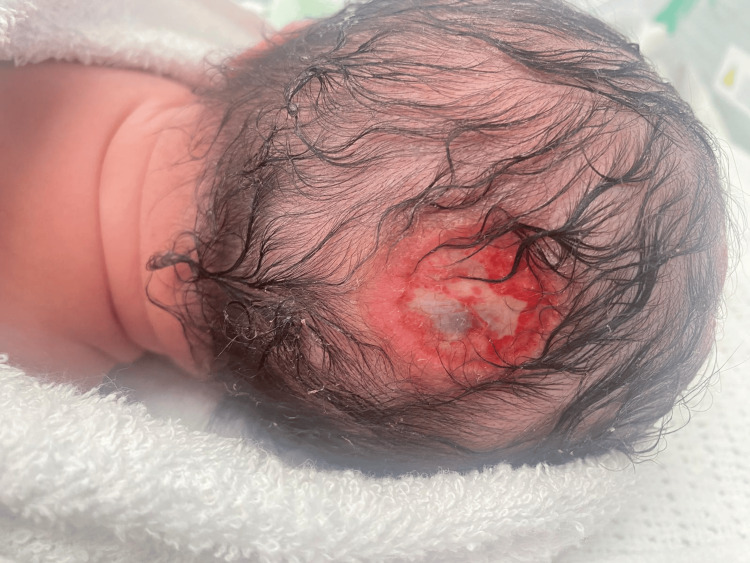
View of the lesion on the back of the infant's head This lesion appeared particularly thin, with visible pulsatile material beneath it.

A newborn male infant was delivered by emergency cesarean section and noted to have extensive defects to his scalp by the attending team. There were ulcerated patches on the top of his scalp (Figure 1), and overlying his occiput was an area with a thin translucent membrane with visible pulsatile material beneath it (Figure 2). There were no other abnormalities on examination. He was admitted to the neonatal unit for observation overnight, as there were concerns that the lesions could be easily damaged. He was nursed in the prone position to help protect the back of his head and given analgesia as he was agitated (the cause of which remains uncertain, whether it was pain due to the lesions or hunger).

**Figure 3:**
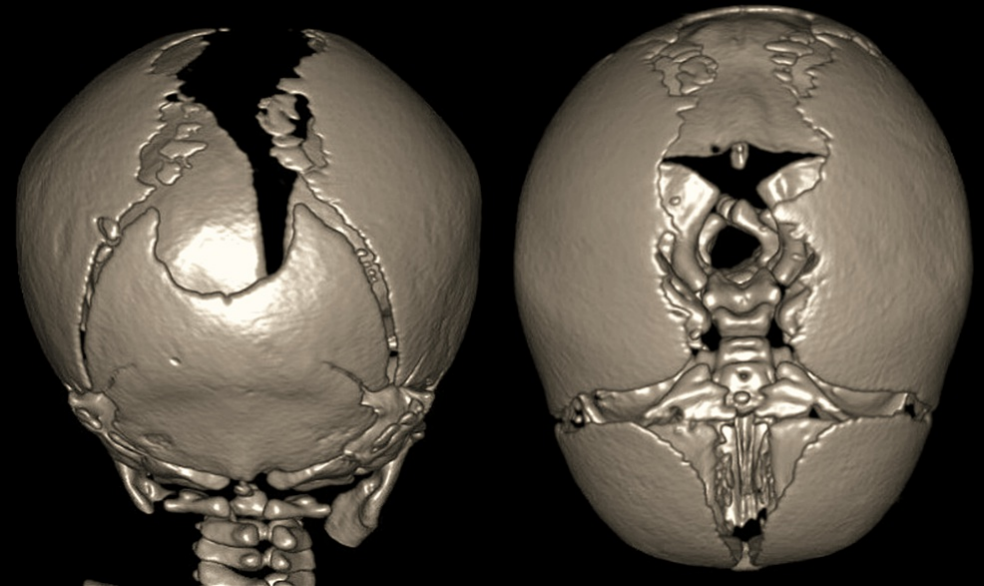
CT scan of the head The 3D reconstruction from a CT scan reveals extensive defects extending posteriorly from the anterior fontanelle.

The following morning, the plastic surgery and neurosurgical teams discussed the case and reached a diagnosis of ACC. The specialist teams advised a cranial ultrasound scan and a CT scan to assess the lesions. The CT scan showed no underlying bone beneath the affected areas (Figure 3).

**Figure 4:**
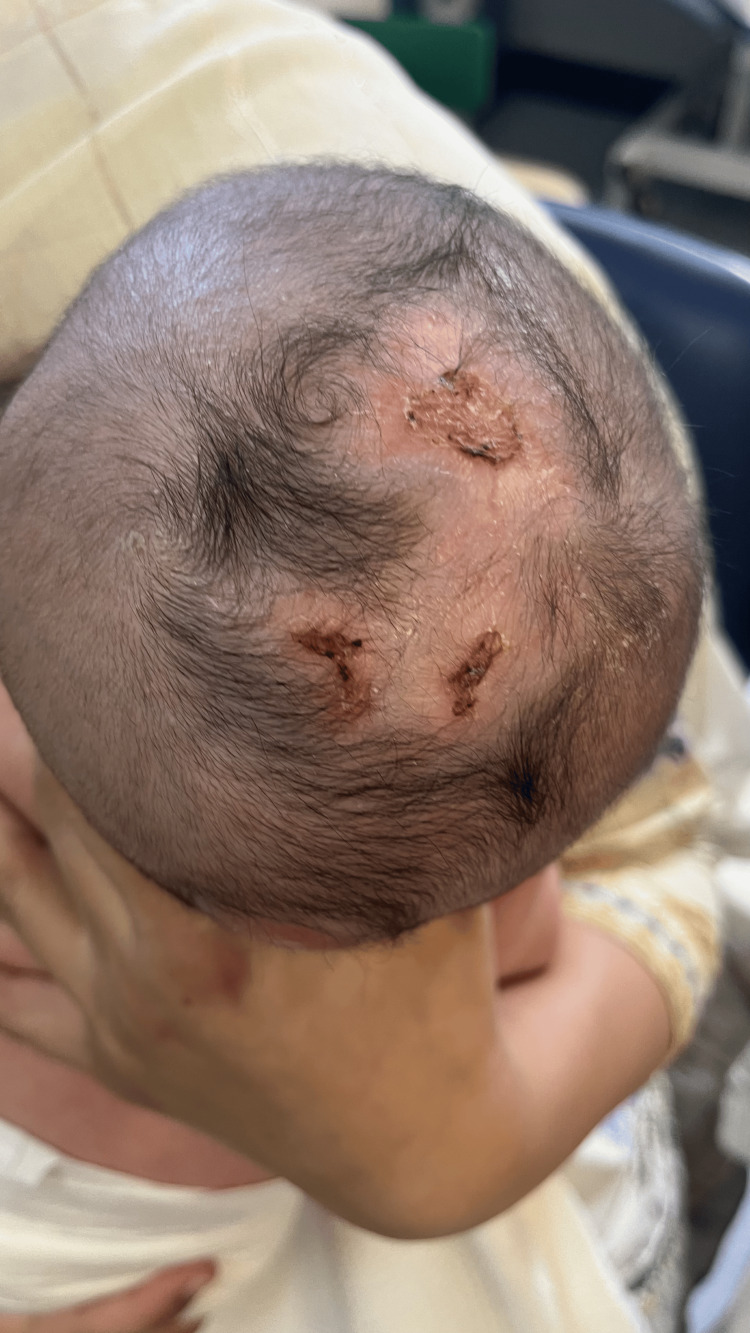
The affected infant at six weeks old At six weeks old, the infant's lesions have a hairless covering and are still soft to the touch, with no underlying bone.

Following further discussion with the surgical teams, he was discharged home for conservative community management, involving safety advice for parents, community nurse input to monitor wound healing and perform dressing changes, and planned neonatal and surgical follow-up. The patient has responded well to this management. The lesions are slowly healing with regular dressing changes and an MRI is planned along with genetic testing as there is a family history of infants being born with similar, although less extensive, defects. At a six-week clinic appointment, the lesions appear to be in a more favorable condition, exhibiting a thin and hairless covering without any underlying bone (Figure 4). He is still under the ongoing supervision of the plastic surgical team, and there are currently no plans to perform surgery to correct the defects.

## Discussion

ACC is a rare congenital absence of the epidermis, dermis, and occasionally subcutaneous tissue. It has an incidence of approximately 1 in 10,000 births [1]. Up to 90% of lesions are localized to the scalp but can affect any area of the skin [2]. Nine described varieties of ACC have been documented (Table 1). These range from solitary lesions to an association with systemic problems such as epidermolysis bullosa or limb abnormalities. It can also be associated with syndromes such as trisomy 13. The pathogenic mechanism is not understood, and there is no unifying genetic disorder identified. It is hypothesized that increased skin tension during fetal development causes ACC and that this could be caused by teratogens, intrauterine ischemia, or intrauterine infection [3]. *BMS1*, a gene involved in fibroblast function, has been implicated in five generations of a family with autosomal dominant ACC, but there are many reports of sporadic mutations in other genes causing the condition [4].

**Table 1:** Types of ACC

ACC Type	Defect
Type 1	Scalp ACC with no other abnormality
Type 2	Scalp ACC with limb abnormality
Type 3	Scalp ACC with epidermal or sebaceous nevus
Type 4	ACC overlying deep embryological malformation
Type 5	ACC associated with fetus papyraceous
Type 6	ACC with epidermolysis bullosa
Type 7	ACC of the extremities only, without blistering
Type 8	ACC associated with an identified teratogen
Type 9	ACC associated with a malformation syndrome

Complications include infection or bleeding, which can be life-threatening if the sagittal sinus is involved. Patients with scalp lesions that become infected are at an increased risk of meningitis and require prompt management. Historically, mortality in individuals with ACC was reported as 20%-55% [5], although more up-to-date figures are likely lower, and some of this reported mortality was due to sagittal sinus bleeding directly due to attempted surgical intervention.

There is no specific consensus on how best to manage lesions. Small lesions are generally managed conservatively, with regular dressings and antimicrobial cream until the defect heals. The missing bone does not regenerate, and the lesions heal to form hairless, atrophic scars. Larger lesions need careful monitoring and assessment for any underlying structural defects and may need surgery such as skin grafting or cranioplasty. However, this surgery is not without its risks as described previously. Generally, lesions with venous involvement, lesions that expose underlying structures, or lesions over a certain size (>15 cm^2^) should be considered for early surgical management such as skin grafting [1].

This case is a Type 1 ACC with large scalp defects. There is evidence in the literature that lesions of this size can be managed either conservatively or surgically. Conservative management for even large lesions (9x10 cm) or multiple lesions such as in this case has been reported to result in complete epithelization of the skin by the age of five [6,7]. Similarly, a case of a 10x5 cm lesion managed surgically with a scalp flap is documented [7]. The team proceeded to surgical management in this case due to the proximity of the lesion to the superior sagittal sinus.

Each case of ACC should be assessed with input from both medical and surgical teams. The size and location of lesions are crucial in determining whether conservative or surgical management is required. However, it is recommended to initially opt for conservative management to see if healing can occur without any intervention.

## Conclusions

While there is no clear guideline for the management of cases such as this, one can suggest a method to approach and evaluate infants born with this condition. It is important to conduct a thorough physical examination, focusing both on the lesions (their size, location, and underlying structures) and the baby as a whole (dysmorphic features or other skin problems).

These lesions can be unpredictable, and there is a risk with scalp ACC of direct trauma to the brain. Admitting the infant to the hospital while the assessment is in progress can provide necessary protection. Early involvement of both plastic surgery and neurosurgery can greatly assist in guiding continuous management. Cranial imaging can provide a more in-depth view of the defects. These lesions can sometimes be managed conservatively, and if this is the case, they will need community input to help with dressing changes and monitoring for complications such as infection. Certain lesions may require surgery for repair, which can be facilitated by local surgical teams and adhering to local medical practice.
